# Stress-sensitive antidepressant-like effects of ketamine in the mouse forced swim test

**DOI:** 10.1371/journal.pone.0215554

**Published:** 2019-04-15

**Authors:** Paul J. Fitzgerald, Jessica Y. Yen, Brendon O. Watson

**Affiliations:** Department of Psychiatry, University of Michigan, Ann Arbor, Michigan, United States of America; Chiba Daigaku, JAPAN

## Abstract

Major depression is a stress-linked disease with significant morbidity and the anesthetic drug ketamine is of growing interest in the treatment of depression, since in responsive individuals a single dose has rapid (within hours) antidepressant effects that can be sustained for over a week in some instances. This combination of fast action and a therapeutic effect that lasts far beyond the drug’s half-life points to a unique mechanism of action. In this reverse translational study, we investigate the degree to which ketamine counteracts stress-related depression-like behavioral responses by determining whether it affects unstressed animals similarly to stressed mice. To test this, male C57BL/6J mice were given a single injection of vehicle (0.9% saline; i.p.), 10 mg/kg ketamine, or 30 mg/kg ketamine, and were tested in the forced swim test (FST) 24 hours and 7 days later, as well as in the open field test on the eighth day. Unstressed mice had normal group housing, environmental enrichment, and experimenter pre-handling (5 days), whereas stressed animals were subjected to chronic mild stress (single housing, reduced enrichment and minimal handling), where some mice also had daily two-week unpredictable chronic stress (UCS). We find that ketamine (24 hours post-injection) decreases immobility and increases mobile (swimming) behavior (antidepressant-like effects) in UCS animals but does the opposite in unstressed mice, similar to recent human findings. In summary, these data suggest that chronic psychological stress interacts with ketamine treatment to modulate its effects in the C57BL/6J mouse FST, which reinforces the relevance of this test, and this strain of mice, to human, stress-induced depression.

## Introduction

Major depression, which in many cases is induced by exposure to ongoing marked psychological stress or trauma, is a major public health problem worldwide [1–3]. Treatment of this debilitating neuropsychiatric disorder is hindered by the delayed response and, in many cases, therapeutic resistance to monoaminergic antidepressants such as the selective serotonin reuptake inhibitors (SSRIs) and tricyclics [4,5]. The response of patients to a single dose of the anesthetic drug ketamine, which gives rapid relief of symptoms in many individuals and outlasts the presence of the drug itself by days to weeks, is fundamentally different from other antidepressive treatments. Infusion of ketamine at a subanesthetic dose indeed has rapid and sustained antidepressant effects in approximately 50% of patients with treatment-resistant depression [6–8]. Interestingly, rodents show a behavioral response to low-dose ketamine on a very similar timescale to that of humans: the response starts within minutes and lasts for days to weeks [9–11]. Translational rodent research originally prompted the discovery of ketamine in human depression [6], and now reverse translational research helps facilitate further innovation in our understanding of major depression and its pharmacological treatment.

Exposure to marked and often chronic psychological stress or trauma is a frequent etiological factor in the induction of major depression in human subjects [12,13], so valid rodent antidepressant treatment models will benefit by exhibiting stress-dependent behavioral responses. A number of studies on the antidepressant-like properties of ketamine have been carried out in rodents in recent years, and while many of these studies report that ketamine has antidepressant-like effects in stressed animals, there are mixed reports on its effects in unstressed controls [9,14–17]. Some of these findings and others suggest that ketamine administration to C57BL/6J mice interacts with psychological stress to modulate antidepressant-related behavior, but other studies report qualitatively similar results in both stressed and unstressed mice.

To study in rodents the mechanisms of this medication with an apparently novel mode of action, it will be important to understand how ketamine interacts with stress-induced behavioral changes. We therefore set out to establish the degree to which the effects of ketamine are stress-dependent using the well-established FST and open field test (OFT) assays to measure ketamine effects in stressed (including UCS) versus unstressed animals from the most commonly-used mouse strain in behavioral neuroscience, C57BL/6J. In this scenario, we feel it is important to not only use a group of mice exposed to chronic psychological stress to investigate depression-like behavior and response to pharmacotherapy, but also include an unstressed group as an experimental control that should not on average be in a depression-like state and may respond differently to antidepressant drugs.

## Materials and methods

### Subjects

One hundred sixty-eight (n = 8 stress/drug cohort; see below) experimentally naïve adult (8–9 weeks old upon arrival) male C57BL/6J mice were obtained from a commercial supplier (The Jackson Laboratory, Bar Harbor, ME). Upon arrival and throughout the experiments, mice were single or group housed (depending on the experiment) in cages within a humidity- and temperature-controlled vivarium, and kept on a 12:12 hr light/dark cycle (lights on at 6 am) with ad libitum access to food and water. All experiments were conducted in the daytime during the light phase. All procedures were carried out at the University of Michigan and were performed in strict accordance with the guidelines and regulations set forth by the National Institutes of Health and the University of Michigan, with full approval from its Institutional Animal Care and Use Committee (Protocol number: PRO00007803).

### Drug

Ketamine hydrochloride (Ketalar, Par Pharmaceutical, Chestnut Ridge, NY) was stored in darkness at room temperature, and for administration was diluted in 0.9% physiological saline solution (vehicle; VEH) and injected intraperitoneally (i.p.), at a volume of 10 ml/kg.

### Stress procedures

**Fig 1:**
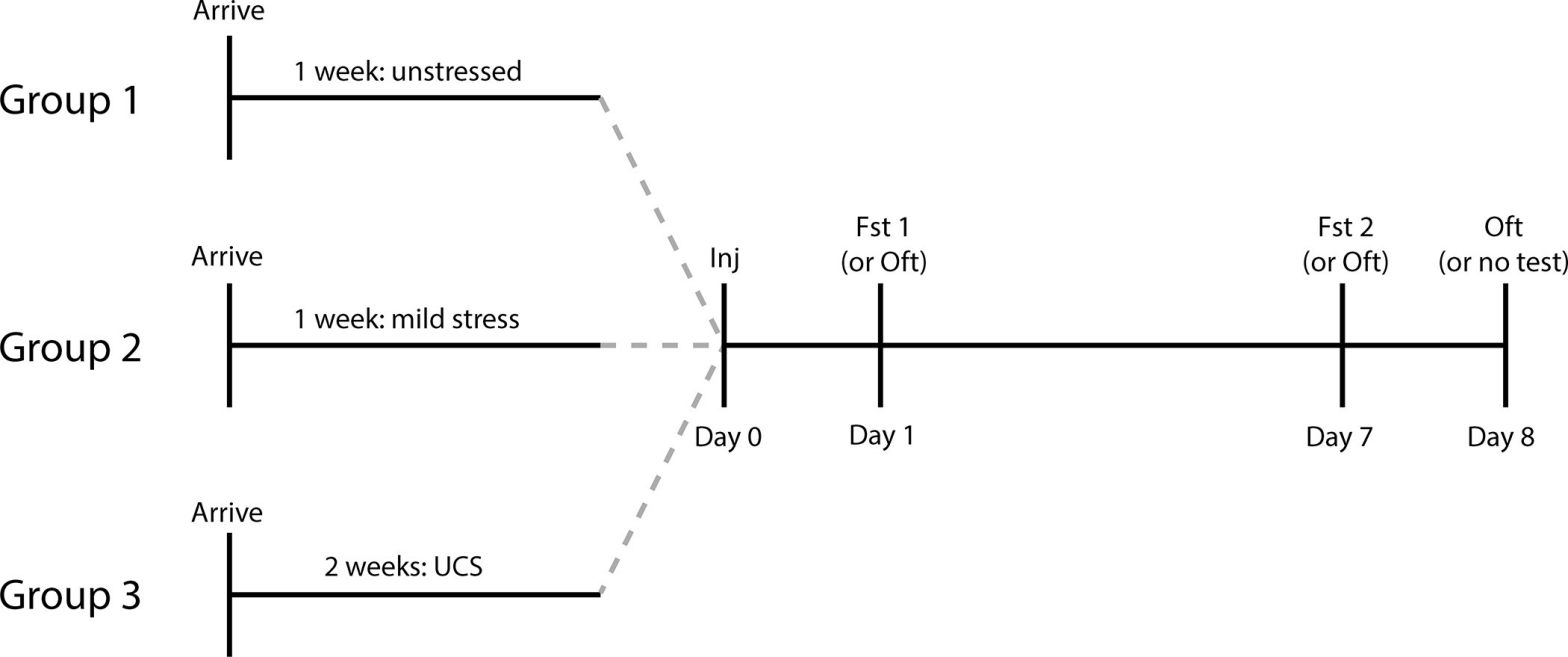
Experimental timeline. Upon arrival at the facility, mice were subjected to either unstressed, mild stress, or unpredictable chronic stress (UCS) conditions, where UCS daily hassles began the next day. Following this period of stress or no stress, all mice received a single injection (Day 0) of either vehicle (0.9% saline, i.p.), 10 mg/kg ketamine, or 30 mg/kg ketamine. 24 hours later (Day 1), mice were given a forced swim test (FST1), followed by a second FST on Day 7 and an open field test (OFT) on Day 8. In additional control experiments, another cohort of mice was injected on Day 0, and given an OFT on Day 1 and on Day 7.

Before forced swim test (FST) or open field test (OFT) experiments began (which were all carried out by author PJF), mice were chronically subjected to one of three stress paradigms: unstressed/standard procedures, mild stress, or unpredictable chronic stress (UCS). Unstressed procedures consisted of group housing of the mice upon arrival in the vivarium and throughout experimentation, handling for ~30 seconds a day for the first five days after arrival, and maintenance of standard levels of cage enrichment with a single package of nesting material (Enviropak, Lab Supply, Fort Worth, TX) instead of nestlets. In contrast, mice subjected to “mild stress” were single housed upon arrival and throughout the experiments, were not handled by the experimenter in the first five days (and throughout the experiments, except when necessary), and their cages were not enriched with an Enviropak and were instead given two white nestlets. A third group was exposed to a stronger stressor, UCS, with the same conditions as “mild stress” plus one stressor/hassle a day at a random time, beginning the day after arrival and lasting for 14 consecutive days (see S1 Table for a description and the sequence of these stressors). These UCS stressors were chosen to be compatible with head cap implanted mice, which may be required in future electrophysiological experiments in our laboratory or other laboratories. The experimental timeline is shown in Fig 1; all mice were weighed two days prior to injection with ketamine or vehicle. All experiments were designed to have 8 mice per stress/drug condition. However, one VEH UCS mouse died before behavioral experiments were carried out.

### Forced swim test (FST)

This is a test of depression-related behavior in rodents [18,19]. Two mice were typically tested simultaneously in a pair of clear Plexiglas cylinders, 30 cm high and 20 cm in diameter, filled halfway with water (24 ± 1 degree C). An opaque plastic divider was placed between the two forced swim tanks to block the animals’ view of one another. Ultrasonic monitoring did not reveal any ultrasonic or sonic-range vocalizations during this testing and so both visual and auditory signaling were unlikely between pair-tested animals. Mice were brought to the testing room in their homecages, and allowed to acclimatize to the room for approximately one hour prior to testing. Overhead, ambient white lighting (600 lux) was present in the room during acclimation and throughout testing. At the start of each trial, the mouse was gently placed in the center of the tank and allowed to swim about freely. Each trial lasted six minutes, but behavior was only scored in the last four minutes [20]. During the test, movement of the animal was tracked with a camera system (mounted horizontally, facing the sides of the two tanks) and software package (EthoVision XT, Noldus Information Technology, Leesburg, VA)–more analysis details below. At the end of the trial, the mouse was immediately removed from the tank, dried off with a paper towel, and returned to its homecage.

Ethovision data were either scored by Ethovision itself and exported as numbers of seconds above varying thresholds (for climbing, swimming and immobility), as was done for most of the paper, or in the case of our raw measures analysis (S4 Fig), we exported raw frame-wise measures. For these raw measures, Ethovision exports an estimated area of frame occupied by the animal and movement is determined by change in the size of that area from one 0.08 sec time bin to the next. Specifically, per-time movement was calculated as: movement = AreaChange/(Area in previous sample + Area in current sample). This movement metric is shown in the histograms in S4 Fig and the mean, median and variance of this quantity were calculated and compared across animal groups.

### Open field test (OFT)

This is a test of anxiety-related behavior and locomotion [21,22] and was carried out 8 days after injection (or 24 hours and 7 days after injection in a locomotion control experiment; see Supplemental Materials, S3 Fig). Two mice were typically tested simultaneously, in neighboring open field boxes that had opaque white Plexiglas walls–again no sonic or ultrasonic vocalizations were detected during initial episodes of this testing paradigm, indicating that these animals do not vocally communicate even when pair-tested. Each box had an enclosed floor and walls, and was cubic with 40 cm long walls and an open top. For analysis, the center region of the box floor was defined offline as a 20 x 20 cm square but was not marked. Each box was placed on the floor of the room, and was illuminated by indirect white lighting from tree lamps to 60 lux as measured by a light meter placed on the floor of the box. At the start of each trial, the mouse was gently placed in a corner of the box, facing the center, and allowed to walk about freely. Each trial lasted 10 minutes and throughout this period, video was recorded for position and movement analysis. The camera was mounted vertically, centered above the two boxes and EthoVision XT software package was used for both acquisition and subsequent analysis (Noldus Information Technology, Leesburg, VA). In this manner, all behavioral activity was recorded automatically with this system. At the end of the trial, the mouse was immediately removed from the box and returned to its homecage. The box was cleaned with 70% ethanol solution and allowed to dry between animals.

### Analysis and statistics

For FST and OFT, movements were assessed automatically using EthoVision software in 80 ms bins (12.5 video frames per second). After analysis of a subset of the FST videos by a trained observer (PJF), we defined “immobile” in EthoVision as comprising bin-by-bin changes in mouse image pixelation of 0–12%. “Mobile” (i.e., swimming) was defined as pixelation changes of 12–18%. “Highly mobile” (i.e., climbing) was defined as pixelation changes greater than 18%. These are also the default settings suggested by Noldus Information Technology. This essentially means that the “highly mobile”, “mobile” and “immobile” classifications are stratifications of degree of movement from frame to frame.

OFT position and movement data were also determined automatically using EthoVision, including quantification of behavior in the center square versus when the animal was near the walls, as described above.

We analyzed the data with conventional parametric statistics (GraphPad Prism, GraphPad Software, La Jolla, CA). Mice showing outlier behavior > 2 standard deviations from the mean for a given FST or OFT test were excluded from the analysis. One-way or two-way analysis of variance (ANOVA) was used to assess general main effects and interactions (stress x drug; α = 0.05). False discovery rate (FDR) analysis and Tukey’s post test were used for subsequent pairwise comparisons of means. Results are shown as mean ± standard error of the mean (SEM). Analysis of mean, median, and variance of raw movement per animal was carried out with EthoVision-assessed movements per frame, without imposed highly mobile/mobile/immobile thresholds.

## Results

We examined how ketamine effects interacted with stress state by comparing automatically-scored FST and OFT behavior after ketamine dosing in cohorts of unstressed, mildly stressed and UCS-stressed mice. We gave vehicle (VEH– 0.9% saline), ketamine at 10 mg/kg (KET10) and 30 mg/kg (KET30) to separate cohorts of animals in each stress group and compared our nine combinations of stress and dose conditions in FST and OFT at multiple time points (Fig 1). There was a small but statistically significant difference in mouse weight after stress but prior to injection with ketamine: unstressed = 26.2 ± 0.3 g (mean ± SEM), mildly stressed = 24.6 ± 0.4, UCS = 26.1 ± 0.3; p < 0.01.

As a positive control to validate antidepressant-like pharmacological responses in our testing environment, we performed the FST in a separate cohort of naïve animals that were acutely injected with the tricyclic antidepressant, desipramine (S1 Fig). We chose desipramine, rather than a selective serotonin reuptake inhibitor (SSRI), since C57BL/6 mice can exhibit behavioral resistance to SSRIs, or instead show depression-like responses to them [23,24]. Desipramine-treated mice exhibited an increase in climbing (highly mobile) behavior in the FST (S1A Fig), when all mice were included in the analysis (t = 2.23, df = 14, p < 0.05), and also when a single > 2 standard deviations outlier was excluded (t = 2.20, df = 13, p < 0.05). Swimming (mobile) behavior was not affected by this drug (S1B Fig, p > 0.05), whereas immobility (S1C Fig) decreased with all mice included (t = 2.30, df = 14, p < 0.05), and showed a statistical trend toward significance with the outlier mouse excluded (t = 2.09, df = 13, p = 0.056). This positive control validates the use of the FST in our environment.

### FST 24 hours post-injection

**Fig 2:**
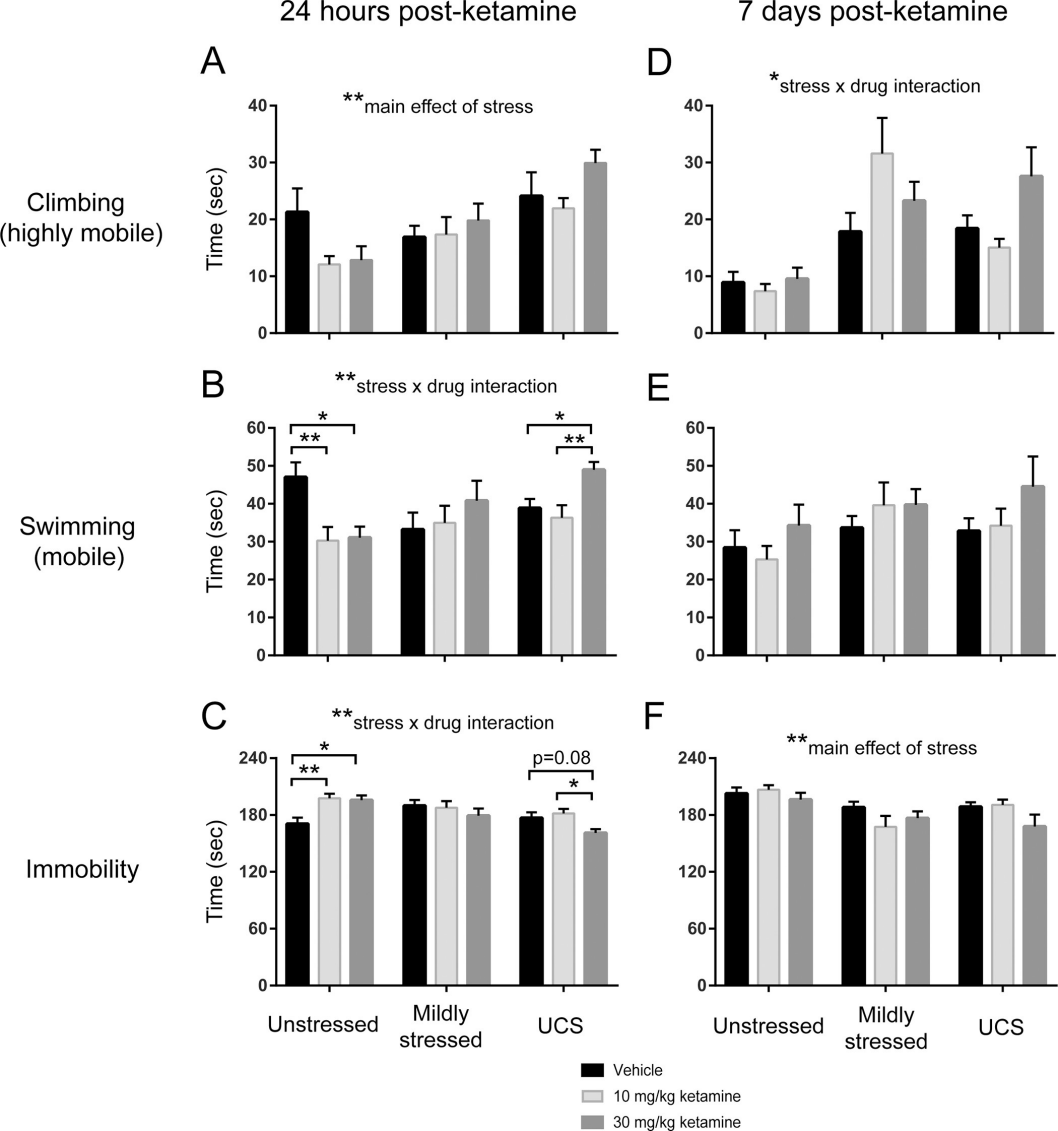
Chronic mild stress and ketamine interact in the mouse forced swim test. Behavior 24 hours post-ketamine was automatically scored in EthoVision and parsed into: **A**, climbing (highly mobile) behavior; **B**, swimming (*intermediately* mobile behavior); **C**, immobile behavior. Swimming behavior in particular revealed differential effects of ketamine according to stress status. Additional swim tests were carried out 7 days post-ketamine: **D**, climbing behavior; **E**, swimming behavior; **F**, immobile behavior. Results trended in the same direction as at the 24 hour time point. Error bars: ± standard error of mean (SEM). Significance indicators for post-hoc tests (horizontal brackets) or two-way ANOVAs (centered over each graph) marked by *p < 0.05, **p < 0.01.

To probe the long-lasting (post-metabolism) effects of ketamine, we carried out the FST 24 hours after ketamine administration, and we specifically wanted to probe how this long-duration effect in mice compared to that in humans wherein depressive state is a key modulator of response. First, we found that prior exposure to stress (especially two-week UCS) was a key variable and increased climbing behavior in FSTs administered 24 hours post-injection [main effect of stress: F(2,57) = 9.47, p < 0.01; Fig 2A], irrespective of ketamine treatment. An analysis of how stress and ketamine interacted showed a stress x drug interaction for swimming behavior 24 hours post-injection: the ketamine groups showed less swimming behavior (indicating a depression-like state) than VEH in the unstressed condition, whereas in the UCS animals KET30 produced increased swimming behavior (antidepressant-like) relative to VEH (and KET10) [F(4,57) = 3.94, p < 0.01; Fig 2B]. While post hoc false discovery rate (FDR) analysis did not reveal any statistically significant pairwise differences in swimming behavior relative to VEH unstressed mice, planned one-way ANOVAs on each stress group did reveal differences within stress groups. Unstressed mice showed differences in swimming behavior with drug [one-way ANOVA: F(2,19) = 7.46, p < 0.01], where Tukey’s post test showed KET10 and KET30 were each significantly lower than VEH (p < 0.01 VEH vs KET10; p < 0.05 VEH vs KET30). Swimming behavior in mildly stressed animals was not modulated by drug treatment (p > 0.05), but it was by UCS [one-way ANOVA: F(2,18) = 6.97, p < 0.01], where Tukey’s post test showed KET30 was significantly higher than VEH (p < 0.05) and KET10 (p < 0.01).

For immobility 24 hours post-injection, there was also a stress x drug interaction, with ketamine increasing (depression-like) immobility relative to VEH in unstressed mice, whereas in UCS mice KET30 produced (antidepressant-like) decreased immobility [F(4,57) = 3.93, p < 0.01; Fig 2C]. Post hoc FDR analysis did not show any significant differences in immobility relative to VEH unstressed mice, but separate one-way ANOVAs on each stress group did show differences with drug treatment. Unstressed mice showed ketamine modulation [one-way ANOVA: F(2,19) = 7.26, p < 0.01]: Tukey’s post test showed KET10 was significantly higher than VEH (p < 0.01), as was KET30 (p < 0.05). Immobility in mildly stressed animals was not modulated by drug treatment (p > 0.05), but it was in UCS mice [one-way ANOVA: F(2,18) = 5.40, p < 0.05], where Tukey’s post test showed KET30 was significantly lower than KET10 (p < 0.05), and KET30 showed a statistical trend toward being lower than VEH (p = 0.081).

Since the pro-depression-like effects of a single injection of ketamine that we report here in the unstressed mice 24 hours post-injection appear to be a novel finding in mice (although not in humans [25]), we carried out a replication experiment in an additional cohort of 24 unstressed mice (S2 Fig). With all 24 replication mice included (S2 Fig, left column), three separate one-way ANOVAs for climbing, swimming, and immobility did not show a significant effect of drug (p > 0.05 in each case), although swimming was decreased by drug without statistical significance. When two mice that were > 2 standard deviation outliers were removed (S2 Fig, middle column), the one-way ANOVA for swimming approached significance [F(2,19) = 2.98, p = 0.075]. We then combined these replication data (with the two outliers removed) with the original unstressed cohort from Fig 2 (which also had its outliers removed) to form a combined group (S2 Fig, right column). This combined group exhibited decreased swimming behavior after ketamine [one-way ANOVA: F(2,41) = 6.84, p < 0.01], and Tukey’s post test showed KET10 was significantly lower than VEH (p < 0.05), as was KET30 (p < 0.01).

### FST 7 days post-injection

Exposure to stress increased climbing behavior in the FST 7 days post-injection [main effect of stress: F(2,59) = 16.09, p < 0.01; Fig 2D], and drug treatment also modulated this behavior [stress x drug interaction: F(4,59) = 2.89, p < 0.05]. Post hoc FDR analysis did not show any significant differences in climbing behavior relative to VEH unstressed mice at this time point, and separate one-way ANOVAs on each stress group also did not show drug differences, although UCS mice showed a statistical trend (p = 0.059) toward being modulated by drug. There were no significant stress or drug effects in swimming behavior 7 days post-injection (Fig 2E). In contrast, stress decreased immobility at this time point [main effect of stress: F(2,59) = 7.72, p < 0.01; Fig 2F], irrespective of ketamine treatment.

### OFT 8 days post-injection

**Fig 3:**
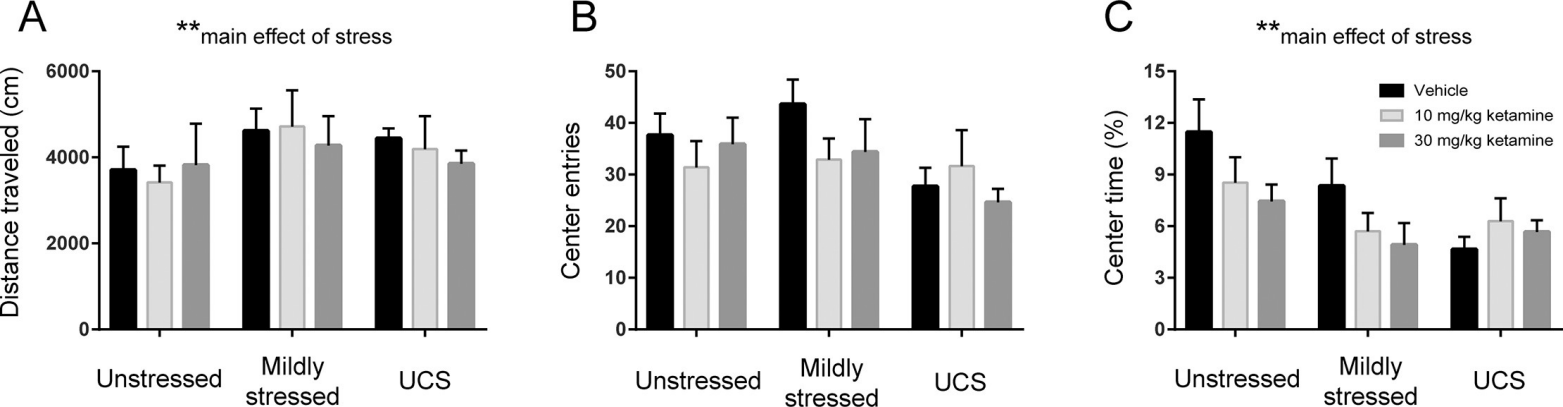
Chronic mild stress but not ketamine modulates the open field test. Behavior (8 days post-injection) was automatically scored in EthoVision and parsed into: **A**, total distance traveled; **B**, center square entries; **C**, percent center square time. There was a main effect of stress, indicating modulation of total distance traveled (*p* < 0.01), as well as percent center square time (*p* < 0.01), with a trend for center square entries (*p* = 0.078). Error bars: ± SEM. Two-way ANOVA ** *p* < 0.01.

Chronic stress exposure, especially in the mildly stressed group, increased total distance traveled in the OFT [main effect of stress: F(2,62) = 11.99, p < 0.01; Fig 3A], independent of ketamine treatment. There was a statistical trend toward stress modulating the number of center square entries in this behavioral test, with UCS mice showing the lowest amount [main effect of stress: F(2,62) = 2.65, p = 0.078; Fig 3B]. Chronic stress also decreased (an anxiogenic-like effect) the percent center square time [main effect of stress: F(2,60) = 6.35, p < 0.01; Fig 3C], without ketamine having a significant effect.

To test whether the behavioral changes observed in the FST at 24 hours and 7 days post-injection could be attributed to general changes in locomotor activity, we performed a set of additional OFT experiments in a separate cohort of naïve mice (S3 Fig). We tested an unstressed group and a UCS one in the OFT at these two time points (without any FST tests in these animals). At 24 hours post-injection, we did not observe any statistically significant effects of drug or stress on three locomotion-related OFT measures: total distance traveled (S3A Fig), center square entries (S3B Fig), or percent center square time (S3C Fig) (each p > 0.05). At 7 days post-injection in these same animals, stress exposure (irrespective of drug) increased total distance traveled [main effect of stress: F(1,40) = 14.73, p < 0.01; S3D Fig] as well as center square entries [main effect of stress: F(1,40) = 4.34, p < 0.05; S3E Fig], but did not alter percent center square time (p > 0.05; S3F Fig). Thus, ketamine did not produce general changes in locomotion at these two time points.

### Behavioral correlations

**Fig 4:**
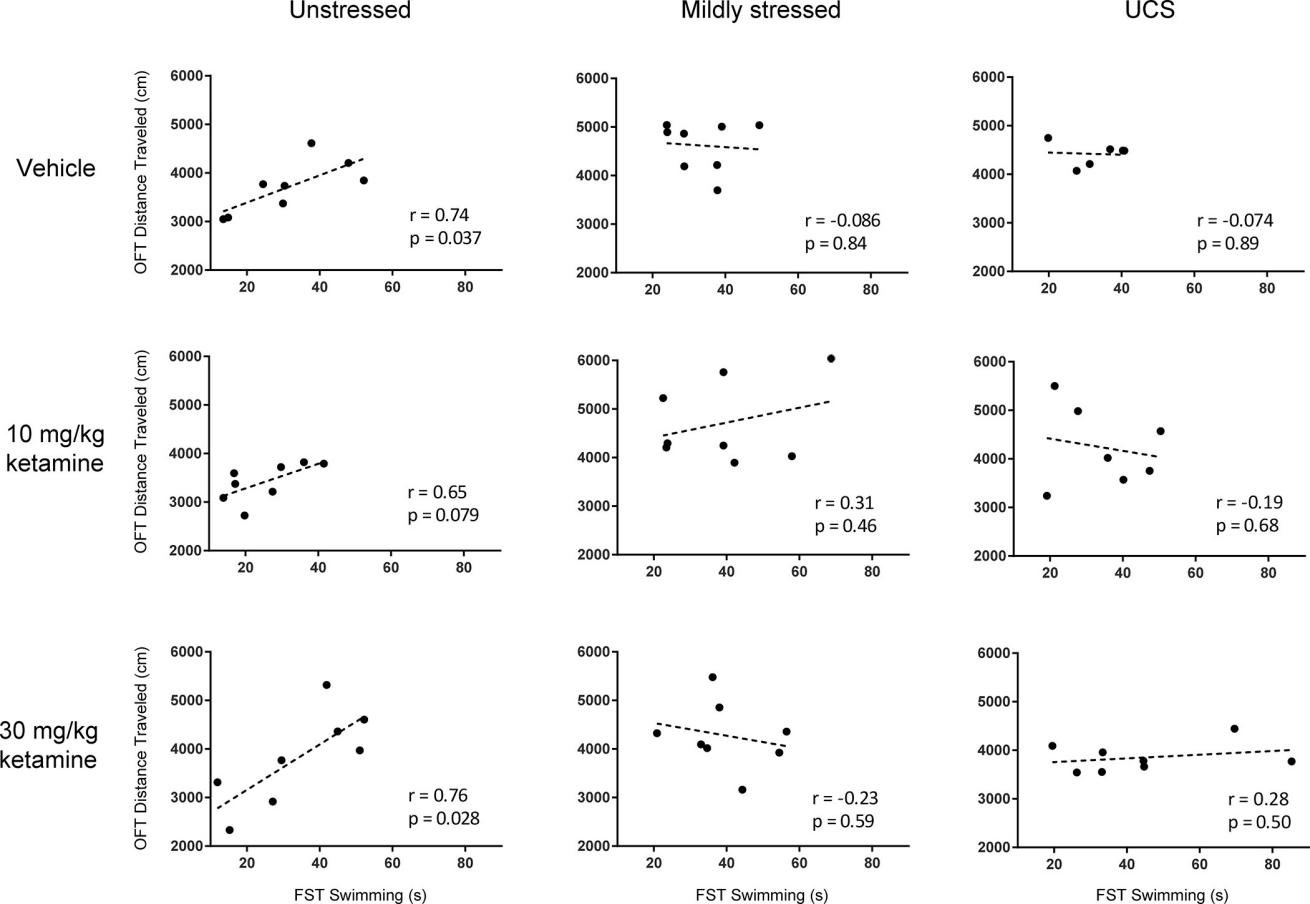
Chronic mild stress disrupts the relationship between FST swimming (mobile) behavior and OFT distance traveled. Each point in each graph represents FST swimming behavior (7 days post-injection, abscissa) versus OFT distance traveled (8 days post-injection, ordinate) for an individual mouse. The correlation coefficient (r) and corresponding p value are shown on each graph. While FST 7 days post-injection swimming and OFT Day 8 distance traveled are correlated in two of three unstressed cohorts, none of the stress cohorts show significant correlation between these metrics.

To further study the behavior of individual mice across the three main tests (FST 24 hrs post-injection, FST 7 days post-injection, OFT 8 days post-injection), we calculated correlation coefficients for pairs of these tests for each animal (parsed by climbing, swimming, immobile in FST; and total distance and percent center time in OFT), sorted by the three stress conditions and three drug treatment groups. This was possible since the same animals were subjected to all three tests and we were interested to see whether individual animal tendencies were apparent across tests and contributed to results. Exploratory analyses revealed a high degree of correlation between FST 7 days post-injection swimming behavior and OFT (day 8) total distance traveled, but only in *unstressed* mice. Fig 4 shows this relatively high degree of positive correlation in unstressed mice, for all drug groups, whereas the six graphs for mild stress and UCS did not show statistically significant correlations (p > 0.45 in each case). FST 7 days post-injection swimming behavior also showed positive correlations with OFT percent center time (Table 1, top), for unstressed VEH and KET10, but not KET30 or any of the stressed groups (p > 0.13 in each case).

**Table 1:** Correlations between FST and OFT behavior.

FST 7 days swimming vs OFT % center time			
	Unstressed	Mildly stressed	UCS
Vehicle	r = 0.74, p = 0.036 *	r = -0.14, p = 0.74	r = 0.68, p = 0.14
10 mg/kg ketamine	r = 0.87, p = 0.0053 **	r = -0.22, p = 0.60	r = -0.41, p = 0.36
30 mg/kg ketamine	r = 0.13, p = 0.77	r = 0.49, p = 0.22	r = -0.46, p = 0.25
FST 7 days immobility vs OFT total distance traveled			
	Unstressed	Mildly stressed	UCS
Vehicle	r = -0.63, p = 0.091	r = 0.13, p = 0.76	r = 0.45, p = 0.37
10 mg/kg ketamine	r = -0.66, p = 0.076	r = -0.21, p = 0.62	r = 0.22, p = 0.64
30 mg/kg ketamine	r = -0.77, p = 0.026 *	r = 0.080, p = 0.85	r = -0.34, p = 0.41
FST 7 days immobility vs OFT % center time			
	Unstressed	Mildly stressed	UCS
Vehicle	r = -0.57, p = 0.14	r = -0.067, p = 0.88	r = -0.47, p = 0.35
10 mg/kg ketamine	r = -0.83, p = 0.011 *	r = 0.24, p = 0.57	r = 0.38, p = 0.41
30 mg/kg ketamine	r = -0.053, p = 0.90	r = -0.56, p = 0.15	r = 0.35, p = 0.40

(*Top*) Correlation between FST swimming (7 days post-injection) and OFT percent center time (8 days post-injection). Shown are the correlation coefficient (r) and corresponding p value, for each cohort (7–8 per stress/drug condition) of mice. (*Middle*) Correlation between FST immobility (7 days post-injection) and OFT distance traveled (8 days post-injection). (*Bottom*) Correlation between FST immobility (7 days post-injection) and OFT percent center time (8 days post-injection).

* *p* < 0.05

** *p* < 0.01.

For FST 7 days post-injection immobility versus OFT total distance, the three unstressed graphs showed significant (KET30) or trends toward significant (VEH; KET10) negative correlations, whereas the stressed mice did not (p > 0.36 in each case) (Table 1, middle). FST 7 days post-injection immobility also showed negative correlations with OFT percent center time for unstressed KET10 but not for the other 8 graphs (p > 0.13 in each case) (Table 1, bottom).

### Raw FST and OFT movement measures

For each mouse, we also carried out analysis of raw FST movement values 24 hours post-injection, based on frame by frame percent changes in pixels occupied by the animal’s body, independent of the above cutoffs we had used to define immobility, swimming, and climbing. Grouping the mice into the nine stress x drug groups, we calculated the following activity values for the distribution of frame-wise body shape changes for each animal in a given FST session: mean, median, and variance. For mean (or average frame-wise change in body shape over frames, see Methods for calculation), there was a main effect of stress [F(2,62) = 4.16, p < 0.05], with the two stress groups (especially UCS) showing a greater amount of movement than unstressed (S4A–S4D Fig). The calculation of median activity did not reveal any significant effects of stress or drug [p > 0.05 in all cases; S4E Fig]. Stress, however, increased the average variance of movement within a single swim, particularly in the UCS animals, yielding a significant main effect of stress [F(2,62) = 15.84, p < 0.01; S4F Fig]. Thus, these additional analyses reinforced the observation that our stress conditions modulated FST behavior–specifically increasing movement variance, whereas they did not reveal additional effects of ketamine treatment.

A similar pattern of raw movement was seen for the FST given 7 days post-injection. For mean measurements, there was a main effect of stress [F(2,62) = 4.68, p < 0.05], with the two stress groups exhibiting a greater amount of movement than unstressed. Median activity did not show a significant effect of stress or drug treatment [p > 0.05 in all cases], whereas the variance of movement was elevated in both stress groups [main effect of stress: F(2,62) = 10.05, p < 0.01].

We also calculated raw movement in the open field test, which was given 8 days post-injection. For mean, there was a main effect of stress [F(2,62) = 3.80, p < 0.05], largely driven by an elevation of movement in the mildly stressed group. A similar pattern to mean was observed for median movement [main effect of stress: F(2,62) = 5.54, p < 0.01]. Variance of movement also showed a main effect of stress [F(2,62) = 7.86, p < 0.01], driven by a marked *decrease* in this measure in mildly stressed animals.

We also explored whether there was a relationship between variance of movement in the FST given 7 days post-injection versus variance in the open field test the following day. There was a statistically significant positive correlation between these two tests for unstressed vehicle mice (r = 0.73, p = 0.041), but for each of the 8 other stress x drug combinations, the correlation did not reach statistical significance (p > 0.14 in each case).

## Discussion

Here we have shown that the effects of ketamine on FST performance depend strongly on the stress state of C57BL/6J mice. Specifically, ketamine (24 hours post-injection) produced depression-like behavior in the FST in unstressed animals, whereas when administered at 30 mg/kg it reduced depression-like behavior in mice subjected to unpredictable chronic stress. These data add to a growing literature that suggests ketamine can buffer against chronic stress in C57BL/6J mice, while also providing the (to our knowledge) novel finding that a single injection of this drug can induce depression-like behavior in mice in the absence of significant stress. Below we discuss several topics related to these findings, ranging from clinical relevance to implications for further experiments.

In a recent clinical study, Nugent et al. (2018) found that whereas administration of a single infusion of ketamine tended to be therapeutic in individuals with treatment-resistant depression, it actually induced depressive symptoms in healthy subjects for up to a day [25]. These clinical findings appear to be similar to the mouse data that we report here and indicate the importance of stressed or depressed states in determining the brain response to ketamine. In particular, chronic stress may be promoting a depression-like state in our C57BL/6J mice which we found to be counteracted by ketamine, whereas our unstressed mice showed increased depression-like behavior (i.e., decreased swimming behavior and increased immobility) when given this drug. These findings suggest that the mouse FST provides translationally relevant information on major depression in humans [19], although the relevance of this test to depression has been questioned [26,27]. The C57BL/6J mouse strain in particular has been widely used to study depression-like behavior induced by exposure to chronic stress, such as chronic social defeat stress [17,28] and UCS [9,29,30], and the findings reported here may reinforce the utility of this strain for this purpose.

In attempting to link rodent and human depression-related findings, it is important to consider the major discrepancies between these species and the methods used to determine affective-related data in them. For example, all mice (including unstressed ones) used in the current study were exposed to a very stressful FST (which is stress test-like) to quantify depression-related behavior, whereas healthy controls in human studies typically do not experience a marked stressor or trauma around the time of testing. These discrepancies and others suggest that comparisons between human and rodent depression-related data should only be made with great caution.

Here we have observed that a single injection of ketamine can produce statistically significant modulation of FST behavior, 24 hours after drug administration, where both swimming (mobile) behavior and immobile behavior were affected by this drug depending on stress condition. We also observed a significant stress by drug interaction for climbing (highly mobile) behavior in the FST, seven days after drug administration. This is consistent with the sustained timecourse of ketamine in human subjects with depression, where the therapeutic effects of drug can last a week or more, often peaking within the first 24–72 hours [7,25]. Autry et al. (2011) and Franceschelli et al. (2015) report behavioral effects in mice lasting at least a week, and Denny and colleagues (Brachman et al. 2016) found greatly sustained (i.e., a number of weeks) behavioral effects of ketamine in mice in various depression- and anxiety-related tests [9–11]. An important question for future human and rodent studies of ketamine is to better document the maximum duration of this drug’s therapeutic effects in individual subjects.

Previous studies of the antidepressant-like properties of ketamine, carried out in a variety of mouse strains, do not report opposing effects of this drug with respect to the presence or absence of chronic stress, and many report antidepressant-like effects of this drug in unstressed animals. Potential sources of variability in this literature include differences in animal strain, ketamine dose, ketamine brand (personal communications), type of test used, and even the gender of the experimenter. For example, the prominent recent study of Zanos et al. (2016) reported an antidepressant-like effect of ketamine in unstressed CD-1 mice in the FST (but did not investigate the FST in C57BL/6J mice in this study) [20]. Since there is a high degree of behavioral (and neural) variability between strains of mice [31,32], including inbred and outbred strains, the findings of Zanos and colleagues may point towards a strain difference in ketamine response, relative to the C57BL/6J findings that we report here. Another prominent study of ketamine, Autry et al. (2011), reports an antidepressant-like effect of this drug (3 mg/kg) across multiple time points in unstressed C57BL/6J mice, with qualitatively similar results in stressed animals [10]. Since we report here the effects of two doses of ketamine, 10 and 30 mg/kg, and find significant differences in the effects of the two doses in the presence of UCS, it is possible that differences in dose reported in various studies play a large role in the behavioral discrepancies reported in the literature with ketamine. A third source of variability in the antidepressant-like properties of ketamine in rodents comprises the various tests used to study the behavior, including forced swim, tail suspension, and sucrose preference. A fourth source of potential variability comprises the gender of the experimenter, where recent reports suggest that this can modulate pharmacological outcomes [33,34].

A number of the published ketamine and stress studies do not report data on ketamine in unstressed controls (for example: [35,36]), and if this had been done more frequently, perhaps the current findings would already be established in C57BL/6J mice. It should also be pointed out that a recent study in unstressed male NIH mice found that subchronic (5 days) administration of 10 mg/kg ketamine, increased immobility time in the FST and tail suspension test [37]; however the repeated administration of drug in that study may modulate locomotor activity, which precludes direct comparison with our single injection findings in unstressed mice. It has already been noted by others that both the strain and stress state of the animal may affect antidepressant-like responses to ketamine, where using animals exhibiting depression-like behavior (rather than only unstressed ones) may be critical in investigating affective-like behavioral responses to ketamine [38]. Hashimoto and Shirayama have also suggested that differences in animal strain and stress conditions across studies may have contributed to discrepancies reported on whether ketamine acts primarily through its metabolite, (2R,6R)-hydroxynorketamine [20,38].

We suggest that the stress procedures used in the current study are mild to moderate, and are more mild than many published stress paradigms. Our stressors nonetheless interacted with ketamine treatment to significantly modulate forced swim behavior, while also altering locomotion and increasing anxiety-like behavior in the open field. Thus, our stressors, such as single housing, lack of experimenter pre-handling, lack of environmental enrichment, and daily (two-week) UCS procedures, can be effective tools for modulating antidepressant-related behavior in the mouse FST. Our UCS procedure, for example, is relatively short in duration (two weeks) and only uses one stress procedure/hassle per day, unlike some studies that use several stressors a day or last for many weeks (e.g., [39]). Our procedures provide two simplified, efficient stress paradigms which can in principle be carried out in a repeatable manner across various laboratories in future studies–and have been shown here to have reliable effects across multiple cohorts (S2 Fig). These procedures could thereby facilitate stress-related research by basic and translational biomedical researchers alike, and would not necessarily be limited to use in depression-related studies only.

We also report here that chronic stress disrupted the degree to which a single animal’s behavior in one task predicts its behavior in another task: the correlations between Day 7 FST and Day 8 OFT shown in Fig 4 were significant in unstressed animals but not in mildly stressed or UCS animals. It is not clear why the apparently baseline (unstressed) correlations of mobility measures within animals may be disrupted by stress. One possibility is that in unstressed animals these two tests are able to measure essentially the same metric–tendency to move or explore rather than be immobile, but under stress the two tests probe different underlying neural processes–as if stressed animals experience these tests more differentially than unstressed animals. Or stress induces hyper-mobility in the OFT but reduced effortful swimming in the FST. Another possibility is that chronic mild stress dysregulates cognitive processing of contextual information [40], resulting in a disruption of normal behavioral patterns of individual mice in the FST and OFT. Whereas unstressed animals that were more active (swimming/mobile behavior) in the FST tended to be more active (total distance traveled) in the subsequent OFT, there were no significant drug effects within or between stress groups in the OFT, suggesting that ketamine is not merely producing generalized hyperactivity that appears to be an antidepressant-like effect in the FST, as more directly shown in the experiments of S3 Fig.

Additionally, our findings of increased variance within the raw mobility behavior of individual animals on the FST and in some cases the OFT, are novel to our knowledge and suggest means by which more information may be extracted from the FST, above and beyond the traditional thresholding methods.

In summary, here we have carried out a reverse translational study of the antidepressant-like properties of ketamine in C57BL/6J mice. We find that the effect of ketamine on FST behavior is modulated by chronic mild to moderate psychological stress, which is affected by factors such as single versus group housing, experimenter handling, environmental enrichment, and daily stressors. While the current findings may suggest that the FST in C57BL/6J mice in particular has translational relevance in fostering greater understanding of major depression in human subjects, further inquiry into the behavioral and physiological basis of these pharmacological effects, in both mice and humans, is required to test this hypothesis.

## Supporting information

S1 Fig Antidepressant-like effect of desipramine as a positive control. We administered 10 mg/kg (i.p.; vehicle is 0.9% saline) desipramine to a total of 16 unstressed mice (8 per drug group) 30 min before a single FST. Behavior was parsed into: **A**, climbing (highly mobile) behavior; **B**, swimming (intermediately mobile behavior); **C**, immobile behavior. The left column of data (“All mice”) shows results from all animals in this new cohort of 16 mice. The right column of data (“No outliers”) replots these 16 mice with one > 2 standard deviation outlier removed from the desipramine group. These results establish that a tricyclic antidepressant (desipramine) has an antidepressant-like effect in our environment, by increasing climbing behavior. Error bars: ± SEM. Significance indicators are for two-tailed unpaired t tests (horizontal brackets) marked by *p < 0.05.
(TIF)

S2 Fig Replication of depression-like effect of ketamine in unstressed mice. FST behavior 24 hours post-ketamine injection was parsed into: **A**, climbing behavior; **B**, swimming behavior; **C**, immobile behavior. The left column of data which represents a replication of the experiment whose results are shown in Fig 2A–2C (“Replic (all mice)”) shows results from all animals in this new cohort of 24 (8 per drug treatment) unstressed mice. The middle column of data (“Replic (no outliers)”) replots these 24 mice with two > 2 standard deviation outliers removed. The right column (“Original + replic”) combines the (“Replic (no outliers)”) with the original cohort of unstressed mice from Fig 2, which also had its outliers removed. These results reinforce the findings from our original cohort and suggest that in unstressed mice, ketamine promotes depression-like behavior (decreased swimming) in the FST, 24 hours post-injection. Error bars: ± SEM. Significance indicators for one-way ANOVAs (horizontal line) or post-hoc tests (horizontal brackets) marked by *p < 0.05, **p < 0.01.
(TIF)

S3 Fig Ketamine does not produce sustained general changes in locomotion in the open field test. We tested a new cohort of 48 mice (8 per drug/stress treatment) in the open field test, 24 hours and 7 days post-injection. Behavior was parsed into 3 groups for each testing timepoint. For 24 hours post-injection: **A**, total distance traveled; **B**, center square entries; **C**, percent center square time. For 7 days post-injection: **D**, total distance traveled; **E**, center square entries; **F**, percent center square time. Error bars: ± SEM. Two-way ANOVA * *p* < 0.05, ** *p* < 0.01.
(TIF)

S4 Fig Raw movement analysis reveals effects of stress in FST 24 hours post-injection. **A**, Distribution of frame-by-frame movements for an unstressed mouse (green) versus a UCS-stressed mouse (blue), both receiving vehicle; **B** Second example of distributions of frame-wise movements for another unstressed mouse and another UCS-stressed mouse (both received vehicle); **C**, Distributions in movements for all unstressed vehicle mice versus all UCS vehicle mice. **D,** Stress alters mean movement in the population of all tested mice (7–8 per drug/stress condition); **E**, no significant effects of stress or drug on median movement; **F**, stress also alters variance of movement. Error bars: ± SEM. Two-way ANOVA * *p* < 0.05, ** *p* < 0.01.
(TIF)

S1 Table List of daily stressors/hassles we used for two week unpredictable chronic stress (UCS) in mice. (DOCX)

## References

[1] Hasin DS, Sarvet AL, Meyers JL, Saha TD, Ruan WJ, Stohl M, et al Epidemiology of adult DSM-5 major depressive disorder and its specifiers in the United States. JAMA Psychiatry. 2018; 10.1001/jamapsychiatry.2017.4602 29450462 PMC5875313

[2] Chen Y, Bennett D, Clarke R, Guo Y, Yu C, Bian Z, et al Patterns and correlates of major depression in Chinese adults: A cross-sectional study of 0.5 million men and women. Psychol Med. 2017; 10.1017/S0033291716002889 27919307 PMC5341494

[3] Schmaal L, Hibar DP, Sämann PG, Hall GB, Baune BT, Jahanshad N, et al Cortical abnormalities in adults and adolescents with major depression based on brain scans from 20 cohorts worldwide in the ENIGMA Major Depressive Disorder Working Group. Mol Psychiatry. 2017; 10.1038/mp.2016.60 27137745 PMC5444023

[4] Derivan AT. Antidepressants: can we determine how quickly they work? Issues from the literature. Psychopharmacol Bull. 1995;31: 23–8. 7675984

[5] Ruhe HG, Huyser J, Swinkels JA, Schene AH. Switching antidepressants after a first selective serotonin reuptake inhibitor in major depressive disorder: a systematic review. J Clin Psychiatry. 2006;67: 1836–55. 17194261 10.4088/jcp.v67n1203

[6] Berman RM, Cappiello A, Anand A, Oren DA, Heninger GR, Charney DS, et al Antidepressant Effects of Ketamine in Depressed Patients. Biol Psychiatry. 2000;47: 351–354. 10686270 10.1016/s0006-3223(99)00230-9

[7] Zarate CA, Singh JB, Carlson PJ, Brutsche NE, Ameli R, Luckenbaugh DA, et al A Randomized Trial of an N-methyl-D-aspartate Antagonist in Treatment-Resistant Major Depression. JAMA Psychiatry. 2006;63: 856–64. 10.1001/archpsyc.63.8.856 16894061

[8] Wilkinson S, Webler R, Katz R, Mesut T, Ostroff R, Sanacora G. S106. Acute and Longer-Term Outcomes Using Ketamine as a Clinical Treatment at the Yale Psychiatric Hospital. Biol Psychiatry. 2018; 10.1016/j.biopsych.2018.02.997 PMC6296748 30063304

[9] Franceschelli A, Sens J, Herchick S, Thelen C, Pitychoutis PM. Sex differences in the rapid and the sustained antidepressant-like effects of ketamine in stress-naïve and “depressed” mice exposed to chronic mild stress. Neuroscience. 2015; 10.1016/j.neuroscience.2015.01.008 25595985

[10] Autry AE, Adachi M, Nosyreva E, Na ES, Los MF, Cheng PF, et al NMDA receptor blockade at rest triggers rapid behavioural antidepressant responses. Nature. 2011; 10.1038/nature10130 21677641 PMC3172695

[11] Brachman RA, McGowan JC, Perusini JN, Lim SC, Pham TH, Faye C, et al Ketamine as a Prophylactic Against Stress-Induced Depressive-like Behavior. Biol Psychiatry. 2016; 10.1016/j.biopsych.2015.04.022 26037911 PMC4633406

[12] Hosang GM, Shiles C, Tansey KE, McGuffin P, Uher R. Interaction between stress and the BDNF Val66Met polymorphism in depression: A systematic review and meta-analysis. BMC Med. 2014; 10.1186/1741-7015-12-7 24433458 PMC3912923

[13] Bonde JP, Utzon-Frank N, Bertelsen M, Borritz M, Eller NH, Nordentoft M, et al Risk of depressive disorder following disasters and military deployment: Systematic review with meta-analysis. British Journal of Psychiatry. 2016 10.1192/bjp.bp.114.157859 26892850

[14] Ma XC, Dang YH, Jia M, Ma R, Wang F, Wu J, et al Long-Lasting Antidepressant Action of Ketamine, but Not Glycogen Synthase Kinase-3 Inhibitor SB216763, in the Chronic Mild Stress Model of Mice. PLoS One. 2013; 10.1371/journal.pone.0056053 23390559 PMC3563541

[15] Dong C, Zhang JC, Yao W, Ren Q, Ma M, Yang C, et al Rapid and Sustained Antidepressant Action of the mGlu2/3 Receptor Antagonist MGS0039 in the Social Defeat Stress Model: Comparison with Ketamine. Int J Neuropsychopharmacol. 2017; 10.1093/ijnp/pyw089 27765808 PMC5408970

[16] jie Zhang W, hua Wang H, dong Lv Y, cai Liu C, yao Sun W, jun Tian L. Downregulation of Egr-1 Expression Level via GluN2B Underlies the Antidepressant Effects of Ketamine in a Chronic Unpredictable Stress Animal Model of Depression. Neuroscience. 2018; 10.1016/j.neuroscience.2017.12.045 29294341

[17] Browne CA, Falcon E, Robinson SA, Berton O, Lucki I. Reversal of stress-induced social interaction deficits by buprenorphine. Int J Neuropsychopharmacol. 2018; 10.1093/ijnp/pyx079 29020387 PMC5793841

[18] Porsolt RD, Bertin A, Jalfre M. Behavioral despair in mice: a primary screening test for antidepressants. Arch Int Pharmacodyn Ther. 1977;229: 327–36. 596982

[19] Cryan JF, Holmes A. Model organisms: The ascent of mouse: Advances in modelling human depression and anxiety. Nature Reviews Drug Discovery. 2005 10.1038/nrd1825 16138108

[20] Zanos P, Moaddel R, Morris PJ, Georgiou P, Fischell J, Elmer GI, et al NMDAR inhibition-independent antidepressant actions of ketamine metabolites. Nature. 2016; 10.1038/nature17998 27144355 PMC4922311

[21] Fitzgerald PJ, Barkus C, Feyder M, Wiedholz LM, Chen YC, Karlsson RM, et al Does gene deletion of AMPA GluA1 phenocopy features of schizoaffective disorder? Neurobiol Dis. 2010; 10.1016/j.nbd.2010.08.005 20699120 PMC2955784

[22] Belzung C, Griebel G. Measuring normal and pathological anxiety-like behaviour in mice: A review. Behavioural Brain Research. 2001 10.1016/S0166-4328(01)00291-1 11682105

[23] Samuels BA, Leonardo ED, Gadient R, Williams A, Zhou J, David DJ, et al Modeling treatment-resistant depression. Neuropharmacology. 2011 10.1016/j.neuropharm.2011.02.017 21356220 PMC3110541

[24] Ihne JL, Fitzgerald PJ, Hefner KR, Holmes A. Pharmacological modulation of stress-induced behavioral changes in the light/dark exploration test in male C57BL/6J mice. Neuropharmacology. 2012 10.1016/j.neuropharm.2011.08.045 21906605 PMC3195838

[25] Nugent AC, Ballard ED, Gould TD, Park LT, Moaddel R, Brutsche NE, et al Ketamine has distinct electrophysiological and behavioral effects in depressed and healthy subjects. Mol Psychiatry. 2018; 10.1038/s41380-018-0028-2 29487402 PMC6111001

[26] Yankelevitch-Yahav R, Franko M, Huly A, Doron R. The Forced Swim Test as a Model of Depressive-like Behavior. J Vis Exp. 2015; 52587 10.3791/52587 25867960 PMC4401172

[27] Commons KG, Cholanians AB, Babb JA, Ehlinger DG. The Rodent Forced Swim Test Measures Stress-Coping Strategy, Not Depression-like Behavior. ACS Chemical Neuroscience. 2017 10.1021/acschemneuro.7b00042 28287253 PMC5518600

[28] Donahue RJ, Muschamp JW, Russo SJ, Nestler EJ, Carlezon WA. Effects of striatal ΔFosB over expression and ketamine on social defeat stress-induced anhedonia in mice. Biol Psychiatry. 2014; 10.1016/j.biopsych.2013.12.014 24495460 PMC4087093

[29] Logan RW, Edgar N, Gillman AG, Hoffman D, Zhu X, McClung CA. Chronic Stress Induces Brain Region-Specific Alterations of Molecular Rhythms that Correlate with Depression-like Behavior in Mice. Biol Psychiatry. 2015; 10.1016/j.biopsych.2015.01.011 25771506 PMC4509914

[30] Elizalde N, Gil-Bea FJ, Ramírez MJ, Aisa B, Lasheras B, Del Rio J, et al Long-lasting behavioral effects and recognition memory deficit induced by chronic mild stress in mice: Effect of antidepressant treatment. Psychopharmacology (Berl). 2008; 10.1007/s00213-007-1035-1 18470507

[31] Hefner K, Whittle N, Juhasz J, Norcross M, Karlsson R-M, Saksida LM, et al Impaired Fear Extinction Learning and Cortico-Amygdala Circuit Abnormalities in a Common Genetic Mouse Strain. J Neurosci. 2008; 10.1523/JNEUROSCI.4904-07.2008 18685032 PMC2547848

[32] Fitzgerald PJ, Whittle N, Flynn SM, Graybeal C, Pinard CR, Gunduz-Cinar O, et al Prefrontal single-unit firing associated with deficient extinction in mice. Neurobiol Learn Mem. 2014; 10.1016/j.nlm.2013.11.002 24231425 PMC4017011

[33] Chapman CD, Benedict C, Schiöth HB. Experimenter gender and replicability in science. Sci Adv. 2018; 10.1126/sciadv.1701427 29349293 PMC5770163

[34] Georgiou P, Zanos P, Jenne C, Highland J, Gerhard D, Duman R, et al Human experimenter sex modulates mouse behavioral responses to stress and to the antidepressant ketamine. Biol Psychiatry. 2018;83: S277 10.1016/j.biopsych.2018.02.715

[35] Maciel AL, Abelaira HM, de Moura AB, de Souza TG, Rosa T, Matos D, et al Acute treatment with ketamine and chronic treatment with minocycline exert antidepressant-like effects and antioxidant properties in rats subjected different stressful events. Brain Res Bull. 2018; 10.1016/j.brainresbull.2017.12.005 29253605

[36] Li N, Liu RJ, Dwyer JM, Banasr M, Lee B, Son H, et al Glutamate N-methyl-D-aspartate receptor antagonists rapidly reverse behavioral and synaptic deficits caused by chronic stress exposure. Biol Psychiatry. 2011; 10.1016/j.biopsych.2010.12.015 21292242 PMC3068225

[37] Suárez-Santiago JE, Briones-Aranda A, Espinosa-Raya J, Picazo O. Agonist E-6837 and antagonist SB-271046 of 5-HT 6 receptors both reverse the depressive-like effect induced in mice by subchronic ketamine administration. Behav Pharmacol. 2017; 10.1097/FBP.0000000000000327 28704275

[38] Hashimoto K, Shirayama Y. What Are the Causes for Discrepancies of Antidepressant Actions of (2R,6R)-Hydroxynorketamine? Biological Psychiatry. 2018 10.1016/j.biopsych.2017.12.007 29409592

[39] Surget A, Wang Y, Leman S, Ibarguen-Vargas Y, Edgar N, Griebel G, et al Corticolimbic transcriptome changes are state-dependent and region-specific in a rodent model of depression and of antidepressant reversal. Neuropsychopharmacology. 2009; 10.1038/npp.2008.76 18536703 PMC2669699

[40] Diamond DM, Campbell A, Park CR, Vouimba RM. Preclinical research on stress, memory, and the brain in the development of pharmacotherapy for depression. European Neuropsychopharmacology. 2004 10.1016/j.euroneuro.2004.09.003 15550347

