# Supplementary figures and images for: Stress-sensitive antidepressant-like effects of ketamine in the mouse forced swim test
Source: PLoS One. 2019 Apr 15;14(4):e0215554. doi: 10.1371/journal.pone.0215554 (PMC6464213; doi:10.1371/journal.pone.0215554)

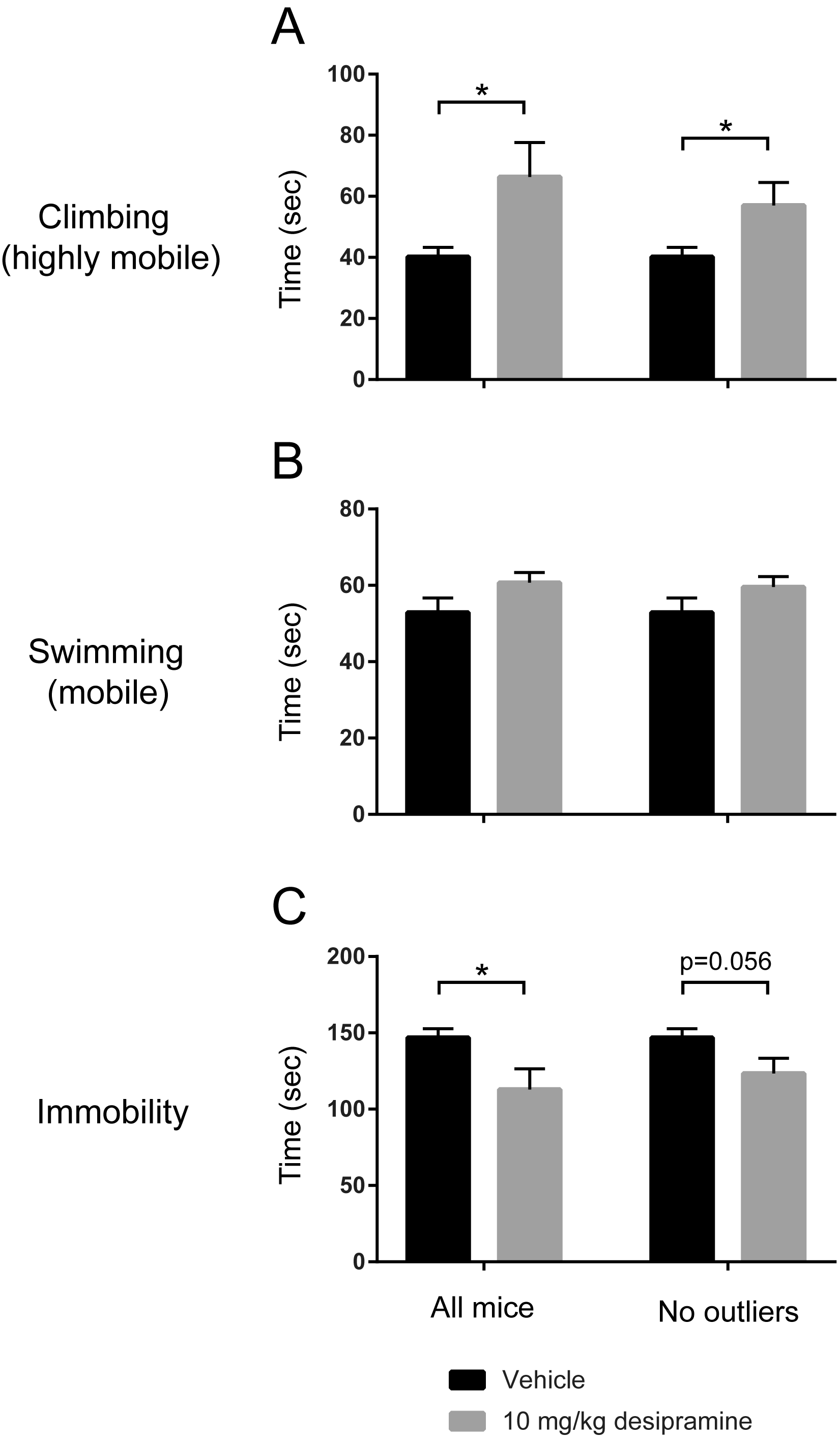

Supplement: S1 Fig — We administered 10 mg/kg (i.p.; vehicle is 0.9% saline) desipramine to a total of 16 unstressed mice (8 per drug group) 30 min before a single FST. Behavior was parsed into: A, climbing (highly mobile) behavior; B, swimming (intermediately mobile behavior); C, immobile behavior. The left column of data (“All mice”) shows results from all animals in this new cohort of 16 mice. The right column of data (“No outliers”) replots these 16 mice with one > 2 standard deviation outlier removed from the desipramine group. These results establish that a tricyclic antidepressant (desipramine) has an antidepressant-like effect in our environment, by increasing climbing behavior. Error bars: ± SEM. Significance indicators are for two-tailed unpaired t tests (horizontal brackets) marked by *p < 0.05. (TIF) [file pone.0215554.s001.tif]

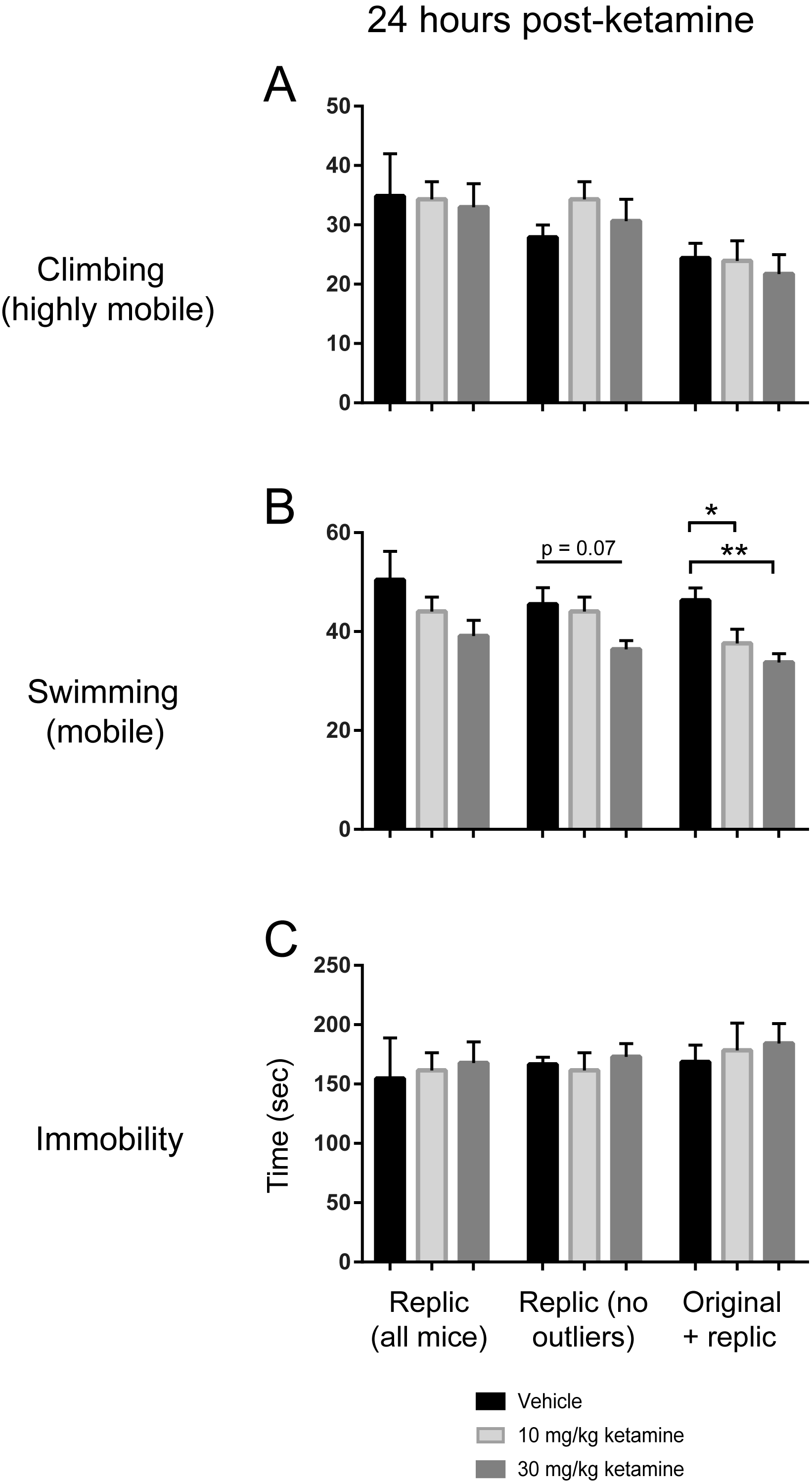

Supplement: S2 Fig — FST behavior 24 hours post-ketamine injection was parsed into: A, climbing behavior; B, swimming behavior; C, immobile behavior. The left column of data which represents a replication of the experiment whose results are shown in Fig 2A–2C (“Replic (all mice)”) shows results from all animals in this new cohort of 24 (8 per drug treatment) unstressed mice. The middle column of data (“Replic (no outliers)”) replots these 24 mice with two > 2 standard deviation outliers removed. The right column (“Original + replic”) combines the (“Replic (no outliers)”) with the original cohort of unstressed mice from Fig 2, which also had its outliers removed. These results reinforce the findings from our original cohort and suggest that in unstressed mice, ketamine promotes depression-like behavior (decreased swimming) in the FST, 24 hours post-injection. Error bars: ± SEM. Significance indicators for one-way ANOVAs (horizontal line) or post-hoc tests (horizontal brackets) marked by *p < 0.05, **p < 0.01. (TIF) [file pone.0215554.s002.tif]

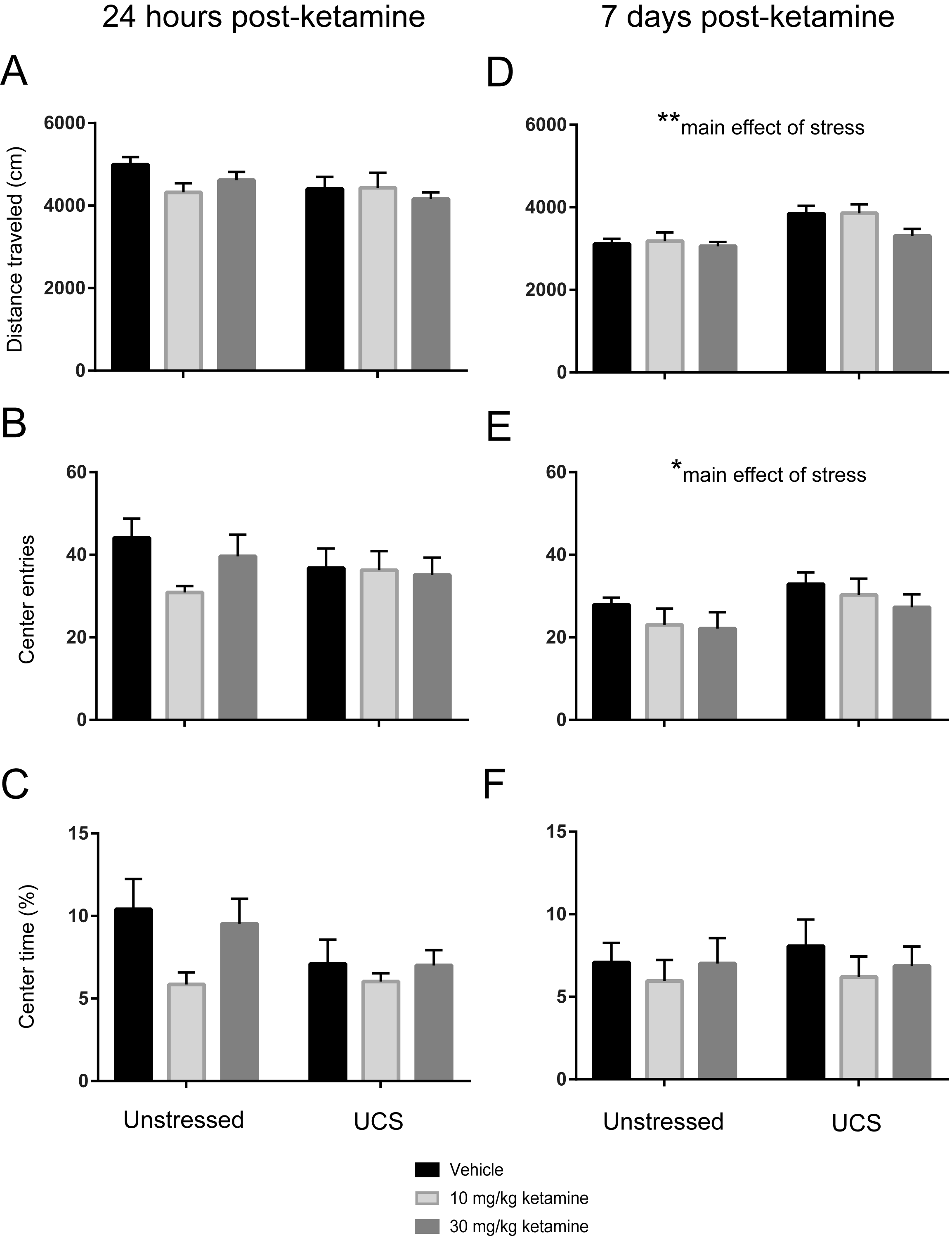

Supplement: S3 Fig — We tested a new cohort of 48 mice (8 per drug/stress treatment) in the open field test, 24 hours and 7 days post-injection. Behavior was parsed into 3 groups for each testing timepoint. For 24 hours post-injection: A, total distance traveled; B, center square entries; C, percent center square time. For 7 days post-injection: D, total distance traveled; E, center square entries; F, percent center square time. Error bars: ± SEM. Two-way ANOVA * p < 0.05, ** p < 0.01. (TIF) [file pone.0215554.s003.tif]

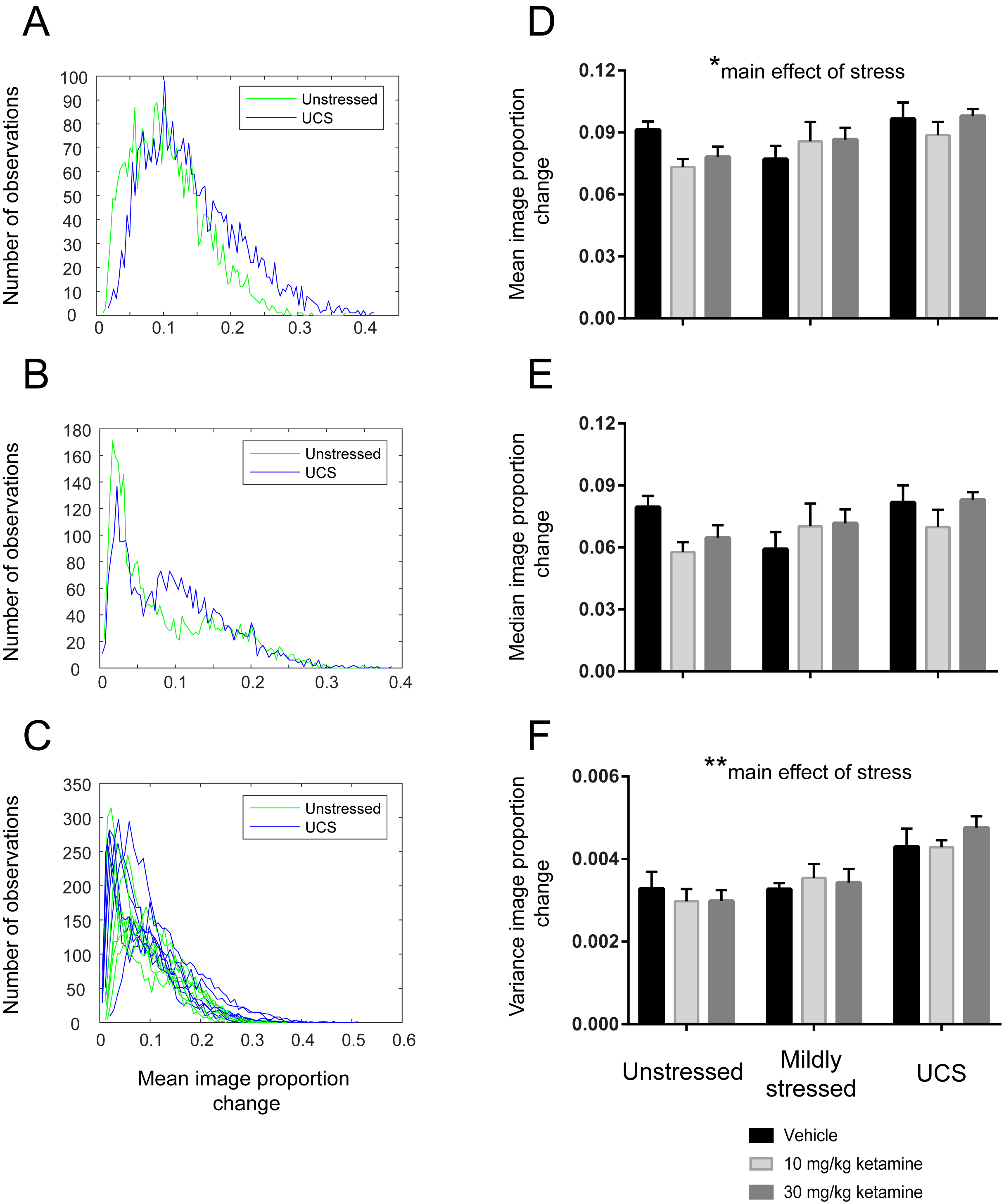

Supplement: S4 Fig — A, Distribution of frame-by-frame movements for an unstressed mouse (green) versus a UCS-stressed mouse (blue), both receiving vehicle; B Second example of distributions of frame-wise movements for another unstressed mouse and another UCS-stressed mouse (both received vehicle); C, Distributions in movements for all unstressed vehicle mice versus all UCS vehicle mice. D, Stress alters mean movement in the population of all tested mice (7–8 per drug/stress condition); E, no significant effects of stress or drug on median movement; F, stress also alters variance of movement. Error bars: ± SEM. Two-way ANOVA * p < 0.05, ** p < 0.01. (TIF) [file pone.0215554.s004.tif]
