# Supplementary material for: Stress-sensitive antidepressant-like effects of ketamine in the mouse forced swim test
Source: PLoS One. 2019 Apr 15;14(4):e0215554. doi: 10.1371/journal.pone.0215554 (PMC6464213; doi:10.1371/journal.pone.0215554)
Supplement: S1 Table — (DOCX) [file pone.0215554.s005.docx]

| Monday | Tuesday | Wednesday | Thursday | Friday | Saturday | Sunday |
| --- | --- | --- | --- | --- | --- | --- |
|  | Mice arrive at facility: immediately single house with only two white nestlets | 9am: Tilting homecage 20° for 2 hrs | Noon: Homecage bedding change, keeping the old nest | 3pm: Homecage change, with 2 new white nestlets | Noon: Turning ambient lights on and off 3 times (1 hr each time) | 3pm: Homecage: removing nest and adding two new white nestlets |
| Noon: Placing mouse in novel empty cage with no bedding, for 1 hour | 9am: Placing divider in homecage for 4 hrs | 3pm: Homecage bedding change, keeping the old nest | 3pm: Turning ambient lights on and off 3 times (1 hr each time) | Noon: Homecage change, with 2 new white nestlets | 9am: Placing mouse in novel empty cage with no bedding, for 1 hour | Noon: Placing divider in homecage for 4 hrs |
| Noon:  Tilting homecage 20° for 2 hrs | 9am:  Homecage: removing nest and adding two new white nestlets |  |  |  |  |  |
